# Prospects and hot spots for mammalian target of rapamycin in the field of neuroscience from 2002 to 2021

**DOI:** 10.3389/fnint.2022.940265

**Published:** 2022-09-01

**Authors:** Lijun Li, Xiaojing Xia, Yunfeng Luo, Yuanting Zhu, Xuhong Luo, Baolin Yang, Lei Shang

**Affiliations:** ^1^Jiangxi Clinical Research Center for Ophthalmic Disease, Jiangxi Research Institute of Ophthalmology and Visual Science, Affiliated Eye Hospital of Nanchang University, Nanchang, China; ^2^Department of Human Anatomy, School of Basic Medicine, Nanchang University, Nanchang, China

**Keywords:** bibliometric analysis, citations, CiteSpace, mTOR, neuroscience, VOSviewer

## Abstract

Mammalian target of rapamycin (mTOR) is an important molecule that regulates cell metabolism, growth, and proliferation in the nervous system. This study aimed to present the current study hot spots and predict the future development trend of the mTOR pathway in neurologic diseases using bibliometrics. We referred to the publications in the Web of Science Core Collection database. VOSviewer and CiteSpace programs were used to evaluate countries/regions, institutions, authors, journals, keywords, and citations showing the current study focus and predicting the future trend of mTOR in neuroscience. The search date ended on 19 June 2022, and there were 3,029 articles on mTOR in neuroscience from 2002 to 2021. Visual analysis showed that although the number of publications declined slightly in some years, the number of publications related to mTOR generally showed an upward trend, reaching its peak in 2021. It had the largest number of publications in the United States. Keywords and literature analysis showed that protein synthesis regulation, ischemia, mitochondrial dysfunction, oxidative stress, and neuroinflammation may be hot spots and future directions of the nervous system in mTOR studies. Recently, the most studied neurological diseases are Alzheimer’s disease (AD), tuberous sclerosis complex (TSC), and depression, which are still worthy of further studies by researchers in the future. This can provide a useful reference for future researchers to study mTOR further in the field of neuroscience.

## Introduction

**FIGURE 1:**
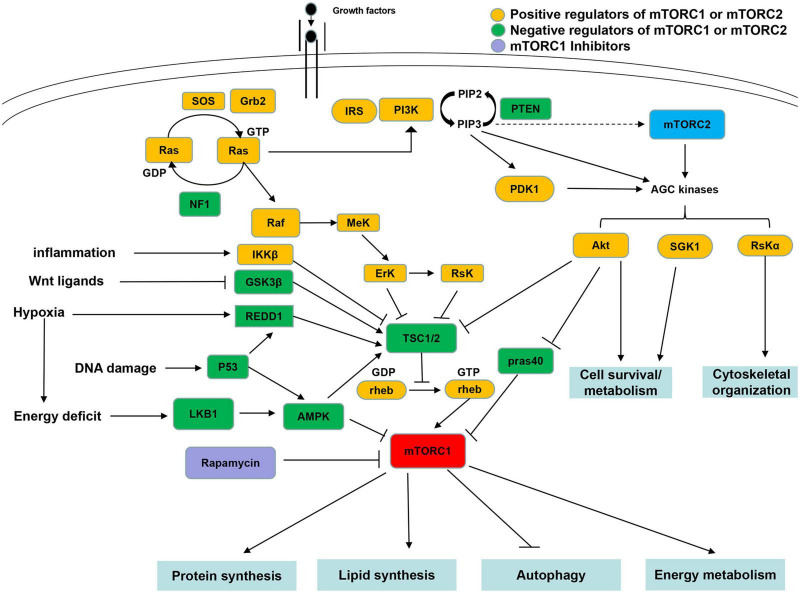
Diagram of the mammalian target of rapamycin (mTOR) signaling pathways. The signaling upstream and downstream pathways of mTOR complex 1 (mTORC1) and mTOR complex 2 (mTORC2). Positive regulators of mTORC1 signaling are shown in yellow, whereas negative regulators are shown in green. mTORC1 and mTORC2 are shown in red and blue colors, respectively. TSC1/TSC2, tuberous sclerosis complex; PI3K, phosphatidylinositol 3-kinase; and AMPK, adenosine 5’-monophosphate-activated protein kinase.

Mammalian target of rapamycin (mTOR), a commonly expressed kinase, is a target of rapamycin and sirolimus (Sehgal et al., 1975; Sehgal, 2003; Benjamin et al., 2011). The discovery of target of rapamycin (TOR) is closely related to the discovery of rapamycin. Rapamycin has gained considerable attention because of its extensive antiproliferative properties. Genetic screening in budding yeast identified TOR1 and TOR2 as mediators of the toxic effects of rapamycin on yeast (Cafferkey et al., 1993; Kunz et al., 1993). Subsequently, scientists purified mTOR by biochemical methods of mammals (Brown et al., 1994; Sabatini et al., 1994; Sabers et al., 1995; Laplante and Sabatini, 2012). As shown in Figure 1, in mammals, mTOR constitutes two catalytic subunits of different complexes: mTOR complex 1 (mTORC1) and mTORC2. mTORC1 controls protein and lipid synthesis, cell growth and proliferation, metabolism, and autophagy, whereas mTORC2 controls cell survival and the cytoskeleton (Laplante and Sabatini, 2012; Ben-Sahra and Manning, 2017; Liu and Sabatini, 2020; Szwed et al., 2021). mTOR signaling pathway plays an important role in obesity (Alemán et al., 2014), insulin resistance (Ardestani et al., 2018), genetic diseases (Li et al., 2014), and type 2 diabetes (Blandino-Rosano et al., 2017; Suhara et al., 2017). In addition, there are an increasing number of studies on mTOR related to the nervous system (Rubinsztein, 2006; Lipton and Sahin, 2014; Crino, 2016).

Bibliometrics is a new method to further determine hot spots in some study fields and has become one of the most useful methods for evaluating the academic influence, centrality, and quality of publications in a certain field, which is significantly important for scientists and those who fund them (Cooper, 2015; Ellegaard and Wallin, 2015; Yan et al., 2021). CiteSpace and VOSviewer are commonly used software packages for bibliometric data analysis (van Eck and Waltman, 2017; Liu et al., 2019).

In this study, we aimed to determine and analyze the global output trend of mTOR in neuroscience using bibliometrics. Data analysis was performed to investigate the current hot spots of mTOR studies in neuroscience and determine future study hot spots. Our analysis provides new insights for researchers to help them plan and manage their scientific work.

## Materials and methods

### Data collection

**FIGURE 2:**
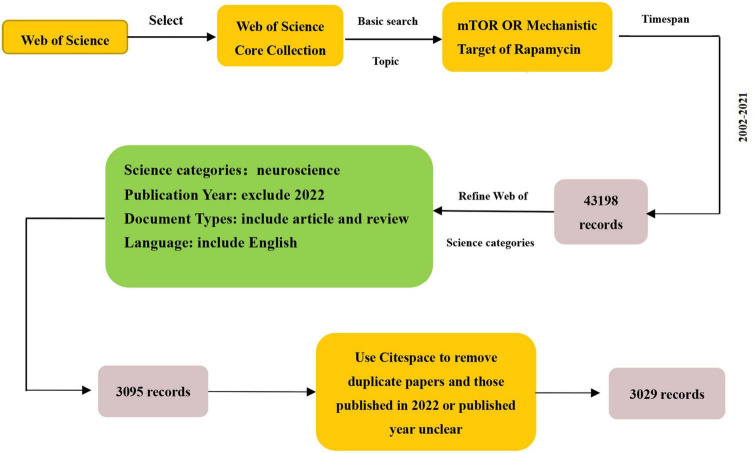
Search flowchart detailing steps in the identification and screening of papers.

We searched the literature related to the Web of Science Core Collection (WoSCC). On 19 June 2022, the keywords “mTOR” or “Mammalian Target of Rapamycin” were used, the time span was 2002–2021, the study direction was neuroscience, the text type was article or review, the language was English, and publications were extracted from the WoSCC. Titles, keywords, author information, abstracts, and references were downloaded in TXT format. Using CiteSpace to remove duplicate papers and papers published in 2022 or with an unclear publication year, 3,029 articles were obtained. The search flowchart is shown in Figure 2. Based on the WOSCC database, we obtained 3,029 documents to analyze the distribution of study fields of countries/regions, institutions, journals, authors, and publications. CiteSpace version 5.6 (R4)^1^ and VOSviewer version 1.6.14^2^ were used to create visualizations and tables. Microsoft Excel 2019 was used to create the tables. The ranking was performed using the standard competition ranking method.

### Co-citation analysis

CiteSpace (version 5.3. R4 64-bit) is a bibliometric program used to identify co-cited authors/references and to capture keywords with strong citation bursts to obtain visual graphs. CiteSpace also analyzed the time trends of the keywords. In total, 3,029 articles were imported into CiteSpace. The ratio k was set to 25, and the retrospective year was set to 1. According to the definition in CiteSpace, each node is a cited paper, and the connection between two nodes represents the cited relationship. The size of the circle indicates the reference file record. The purple circle indicates the central position of the file.

### Data analysis

We selected keywords and key references to predict study prospects and hot spots. The analysis of keywords and key references was performed using VOSviewer and CiteSpace. High-frequency terms, such as key molecules and primary diseases, were used to predict epidemic research disease models and star molecules. The VOSviewer analysis method used was Linlog/modularity, and the CiteSpace analysis method used was log-likelihood. The weights included citations or occurrences. The score represented the average publication year. The thickness of the line indicated the strength of the relationship. The color indicated the average year of publication. Using VOSviewer and CiteSpace software, we analyzed the references, obtained visualization charts and tables, and then analyzed the study direction and hot spots of mTOR in neuroscience.

## Results

### Distribution of publications by year

**FIGURE 3:**
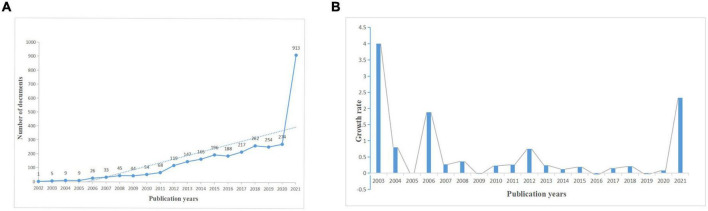
Distribution of publications by year. **(A)** The distribution of publications according to year. **(B)** The growth rate of publications according to year.

Figure 3A shows the time distribution of the number of publications on mTOR in neuroscience. The column diagram shows that the growth rate of the number of articles is increasing (Figure 3B), and the number of annual publications ranges from 1 in 2002 to 913 in 2021 (Figure 3A). Of the 3,029 studies, 266 studies were reviews and the rest were articles. These publications were cited 136,051 times, and each paper has been cited at an average of 44.9 times. This indicates that mTOR has gained increasing attention in the global neuroscience field and may become a study hot spot in the future.

### Countries and regions

Research teams in 41 countries and regions have published 3,029 articles. Some publications were written by researchers from different countries, but we classified them according to country and region. Table 1 lists the top 10 most productive countries and regions. The top 10 most productive countries published 2,271 articles (75%). The United States (*n* = 862, 28.46%) had the highest productivity, followed by China (*n* = 575, 18.98%), Germany (*n* = 139, 4.59%), Italy (*n* = 129, 4.26%), and Canada (*n* = 111, 3.26%). Citation was the most commonly used tool for determining the national efficiency in a specific field. The citation rate in the United States (*n* = 42,599) was significantly higher than that in China (*n* = 9,734).

**TABLE 1:** Top 10 most productive countries and regions with publications on mammalian target of rapamycin (mTOR) in the field of neuroscience.

Rank	Country/region	Documents	Citations	Total link strength	Centrality
1^st^	United States	862	42,599	465	0.06
2^nd^	China	575	9,734	228	0
3^rd^	Germany	139	3,951	222	0.12
4^th^	Italy	129	3,596	209	0.23
5^th^	Canada	111	4,936	116	0.12
6^th^	England	107	4,971	222	0.12
7^th^	Japan	105	4,342	130	0.06
8^th^	France	98	3,714	183	0.28
9^th^	Spain	76	2,193	121	0.28
10^th^	South Korea	69	1,344	33	0

**FIGURE 4:**
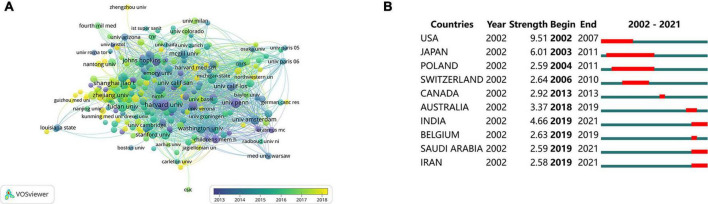
Contributions of countries/regions to mammalian target of rapamycin in neuroscience studies. **(A)** Organizations with co-occurrence relations shown as an overlay graph plotted using VOSviewer 1.6.14. **(B)** Countries/regions with the strongest citation bursts (strong citation bursts indicate that a variable has changed dramatically in a short time; the red bar indicates the duration of the outbreak).

The CiteSpace software was used to analyze the top 10 countries and regions with the strongest citation outbreak (Figure 4B). The United States had the highest blasting strength of 9.51. The outbreak lasted from 2002 to 2007, indicating that more researchers are studying mTOR in China from 2002 to 2007. Israel had the lowest score (2.17). The mTOR study began in 2007 and ended in 2009. From 2019 to 2021, the blasting intensity increased in India, Saudi Arabia, Iran, and other countries, which suggests that related studies on mTOR in neuroscience have attracted the attention of researchers in these countries.

### Organizations

A total of 2,143 organizations published articles, of which 249 reached the threshold. The VOSviewer software was used to analyze the citation networks between these institutions. The top 10 institutions with the most published articles are listed in Table 2. The most prolific institution was Harvard University (*n* = 43, 1.42%), followed by Fudan University (*n* = 32, 1.06%), and Shanghai Jiao Tong University (*n* = 31, 1.02%). Among the top 10 institutions (Table 2), five were American, three were Chinese, and the other two were Canadian and Dutch institutions. However, the total number of citations of these three Chinese organizations (*n* = 1,662) was far lower than that of Harvard University (*n* = 4,435). Figure 4A shows the visual network relationships between these organizations and institutions. Notably, the organizations with the highest citation rates included Harvard University, the University of Pennsylvania, and Columbia University. All of these universities were located in America. This evidence indicates that the United States still dominates mTOR studies in neuroscience.

**TABLE 2:** Top 10 most productive organizations.

Rank	Organizations	Country	Documents	Citations	Total link strength
1^st^	Harvard University	United States	43	4,435	1,073
2^nd^	Fudan University	China	32	683	226
3^rd^	Shanghai Jiao Tong University	China	31	567	193
4^th^	University of Pennsylvania	United States	30	2,003	655
5^th^	Johns Hopkins University	United States	29	1,042	340
5^th^	Zhejiang University	China	29	412	160
7^th^	University of Amsterdam	Netherlands	28	1,210	677
8^th^	Washington University	United States	27	1,900	527
9^th^	Columbia University	United States	26	2,956	426
9^th^	McGill University	Canada	26	1,420	500

### Journals

A total of 3,029 papers were published in 730 Science Citation Index-E journals, of which 134 reached the threshold. Analyzing the distribution of publication sources is helpful to identify the core journals. According to the VOSviewer software analysis, the top 11 published journals (Table 3) were identified. The *Journal of Neuroscience* (*n* = 129, 4.26%), *Molecular Neurology* (*n* = 82, 2.71%), and *Neuropharmacology* (*n* = 68, 2.24%) were the three journals with the largest number of published papers. Five of these top-ranked journals were established in the United States. The journal with the most published articles was the *Journal of Neuroscience*, and the journal with the least published articles was the *Brain Research* (*n* = 47, 1.55%). The impact factors (IFs) of these journals ranged between 3.046 and 6.167. The *Journal of Neuroscience* had the highest IF (*IF* = 6.709) in 2021. Although the number of articles in the *Neuroscience Letters* (*n* = 67, 2.21%) ranked in the top four, its IF was the lowest.

**TABLE 3:** Top 12 largest number of publications.

Rank	Journals	Documents	Country	2022 impact factor
1^st^	Journal of neuroscience	129	United States	6.709
2^nd^	Molecular neurobiology	82	United States	5.682
3^rd^	Neuropharmacology	68	England	5.273
4^th^	Neuroscience letters	67	Netherlands	3.197
5^th^	Journal of Physiology-London	58	England	6.228
6^th^	Journal of neurochemistry	57	England	5.546
7^th^	Neurobiology of disease	54	United States	7.046
8^th^	Frontiers In Cellular Neuroscience	51	Switzerland	6.147
9^th^	Experimental neurology	49	United States	5.62
9^th^	Neurochemical research	49	United States	3.996
11^th^	Brain research	47	Netherlands	3.61

### Authors

**FIGURE 5:**
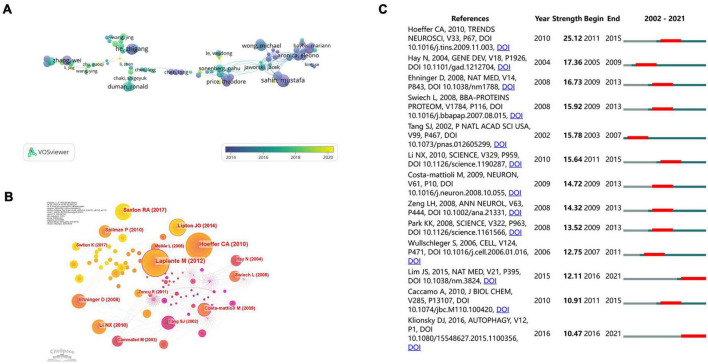
Collaboration and citation networks among core authors and institutions. **(A)** Overlay visualization of co-authorship analysis of authors using VOSviewer. **(B)** Co-citation analysis of references using CiteSpace. **(C)** Top 13 references with the strongest citation bursts based on CiteSpace.

Among the 3,029 papers, there were 17,274 authors in total; 168 authors reached the threshold, and the average number of researchers per paper reached 5.7. Our study revealed cooperation between the most-cited authors. In addition, VOSviewer and CiteSpace analyzed the co-authors and citation networks among the authors. Table 4 lists the 10 core authors of the literature on mTOR in neuroscience from 2002 to 2021. Although the number of articles by Mustafa Sahin was not the highest, its total number of citations (*n* = 1,660) ranked first, which shows that Mustafa Sahin has made great contributions to the study of mTOR in the field of neuroscience. Zhang Wei had the highest H-index (114). Eight of the top 10 core authors were from the United States, and the other two were from Canada and the Netherlands, which indicates that the main authors are from prolific developed countries. In Figure 5A, the size of the nodes represents the number of citations, the connection of nodes represents the collaboration between authors, and the color of the circles represents the average publication year. From the visual map, influential authors, such as Aronica, Eleonora, Kwiatkowski, and David, cooperated more closely. Although Sahin and Mustafa had the highest citation counts, they had less cooperation with other authors. The cooperation between these influential authors was not significantly close, indicating that it needs to be further strengthened in the future. In addition, some emerging study groups were engaged in mTOR studies.

**TABLE 4:** Core authors of publications of mTOR in the field of neuroscience from 2002 to 2021.

Author	Organizations	Documents	Citations	H-index
Sahin, Mustafa	Harvard University (United States)	19	1,660	59
Klann, Eric	New York University (United States)	11	1,554	71
Hoeffer, Charles	University of Colorado Anschutz Medical Campus (United States)	5	1,202	25
He, Zhigang	Harvard Medical School (United States)	7	1,199	45
Phillips, Stuart	McMaster University (CAN)	7	1,046	90
Wong, Michael	Washington University (United States)	14	956	34
Aronica, Eleonora	University of Amsterdam (NL)	22	853	83
Zhang, Wei	Vanderbilt University (United States)	5	806	114
Crino, Peter B.	University of Maryland Baltimore (United States)	19	781	59
Kwiatkowski, David	Harvard Medical School (United States)	8	780	10

### Keywords

**FIGURE 6:**
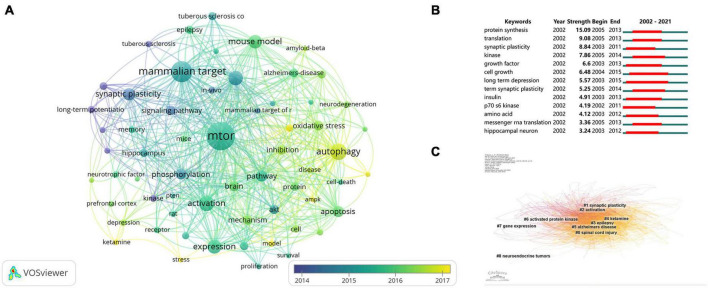
Keywords linked to mammalian target of rapamycin (mTOR) in neuroscience studies. **(A)** The co-occurrence analysis of keywords using VOSviewer. **(B)** Top 13 keywords with the strongest citation bursts on CiteSpace (strong citation bursts indicate that a variable has changed dramatically in a short time; the red bar indicates the duration of the outbreak). **(C)** The clustering analysis of an mTOR co-citation network with keywords based on CiteSpace.

A total of 8,620 keywords were retrieved from the 3,029 documents. To highlight important keywords, we set the minimum number of keyword occurrences to 61, and 61 keywords reached the threshold. The visual network map showed the co-occurrence relationship of keywords (Figure 6A). The size of the circle represents the number of keyword occurrences, and the color of the circle represents the average publication year. As shown in Figure 6A, high-frequency keywords were mTOR, autophagy, activation, and rapamycin. The average publication year for these keywords was 2014. Figure 6B shows the top 13 keywords with the highest number of citations. Protein synthesis showed the highest blasting strength (15.09). The disease of long-term depression continued to explode from 2003 to 2015, indicating that studies on long-term depression, a nervous system disease, have been paid attention to, and it had a close relationship with mTOR, which indicates that this disease will still be a hot spot for researchers in the future. As shown in Figure 6B, from 2015 to 2021, the top 13 keywords with the strongest citations included protein synthesis, messenger RNA, and translation, indicating that the molecular regulation mechanism of mTORC1 will be the focus of the future studies. According to the statistics and classification analysis of keywords, we found that many molecules participated in the progress of mTOR studies. These molecules suggested that some receptors also played a role in mTOR, and different molecules appeared in different cell types. We listed the main molecules, receptors, and cell types (Table 5), which help us to infer the signaling pathways favored by researchers and related star cell types. Table 6 shows the main diseases and pathologies involved in mTOR studies on the nervous system. Currently, the most studied neurological diseases are Alzheimer’s disease (AD), tuberous sclerosis complex (TSC), and depression. The most common pathological mechanisms related to mTOR were oxidative stress, ischemia, mitochondrial dysfunction, neurodegeneration, and neuroinflammation. Figure 6C shows the temporal characteristics of the study fields reflected by the clusters. There were nine clusters, such as cluster 0 (SCI), cluster 1 (synaptic plasticity), cluster 2 (activation), cluster 3 (epilepsy), cluster 4 (ketamine), cluster 5 (AD), cluster 6 (activated protein kinase), cluster 7 (gene expression), and cluster 8 (neuroendocrine tumors). This indicates that mTOR studies in the field of neuroscience are mainly concentrated in these nine directions.

**TABLE 5:** Top 7 key molecules, cell types, and receptor types involved in mTOR in the nervous system.

Rank	Key molecules	Occurrence	Cell types	Occurrence	Receptor types	Occurrence
1	mtor	856	Neuron	131	NMDA receptor	44
2	akt	160	Microglia	49	AMPA receptor	30
3	Kinase	98	Astrocyte	29	Metabotropic glutamate receptor	19
4	PTEN	67	Stem-cells	26	Growth-factor receptor	10
5	BDNF	62	Neural stem-cells	21	Cannabinoid receptor	8
6	AMPK	61	Progenitor cells	20	Glutamate receptor	8
7	Tau	48	Pc12 cells	15	5-ht1a receptor	6
8	mTORC1	39	Cancer-cells	14	D-aspartate receptor	6

**TABLE 6:** Diseases and pathologies involved in the study of mTOR.

Pathology	Occurrence	Diseases	Occurrence
Oxidative stress	168	Alzheimer’s disease	121
Neurodegeneration	88	Tuberous sclerosis complex	121
Neuroinflammation	44	Tuberous sclerosis	90
Axon regeneration	43	Depression	89
Cerebral ischemia	34	Parkinson’s disease	67
Endoplasmic-reticulum stress	32	Autism	54
Hypoxia	28	Temporal-lobe epilepsy	51
Mitochondrial dysfunction	26	Stroke	50
Focal cerebral ischemia	22	Focal cortical dysplasia	49
Ischemia	22	Epileptogenic	39
Ischemia-reperfusion injury	17	Fragile-x-syndrome	37
Axonal regeneration	13	Spinal cord injury	36
Mitophagy	12	Brain-injury	34
Artery occlusion	12	Cognitive impairment	27
Neuronal cell-death	11	Schizophrenia	24
Chronic mild stress	9	Amyotrophic-lateral-sclerosis	22
Hypoxia-ischemia	9	Multiple sclerosis	18
Cerebral-artery occlusion	7	Autism spectrum disorders	7

### Citations

Using VOSviewer to analyze 3,029 references, we listed the top 10 highly cited documents (Table 7). The number of citations ranged from 439 to 811. “Autophagy induction and autophagosome clearance in neurons: relationship to autophagic pathology in Alzheimer’s disease” ranked first in the total number of citations. This article was published by Barry Boland in 2008 and was cited 811 times. Figure 5C shows the top 13 references with the strongest citation burst. The article “mTOR signaling: At the crossroads of plasticity, memory and disease,” published by Charles A. Hoeffer in 2010, had the strongest citation burst from 2011 to 2015. The article “Control of Dendritic Arborization by the Phosphoinositide-3’-Kinase–Akt–Mammalian Target of Rapamycin Pathway” ranked last, with 439 citations. Notably, “Loss of mTOR-dependent macroautophagy causes autistic-like synaptic pruning deficits” ranked fifth, but it has been cited 599 times in the past 7 years. This study paper has been cited many times because it summarizes that mTOR plays an important role in the pathogenesis of autism spectrum disorders. Apart from citation analysis, co-citation analysis was also an important method to evaluate core references. As shown in Figure 5B, the most cited references included Tang et al. (2002), Hay et al. (2004), Ehninger et al. (2008), Li et al. (2010), Hoeffer et al. (2010), and Laplante et al. (2012). Laplante et al. (2012) had the highest centrality, indicating that it is the most influential mTOR in neuroscience. Laplante et al. published a review entitled “mTOR signaling in growth control and disease” in the top journal *Cell*, summarizing the latest 10-year progress of the role of mTOR signaling pathway in health, disease, and aging and the relationship between mTOR and treatment methods related to human diseases. This provides useful knowledge background for scholars who study mTOR signal pathway in neuroscience.

**TABLE 7:** Top 10 highly cited documents of mTOR in the field of neuroscience.

Rank	Title	First author	Journals	Publication year	Total citations
1^st^	Autophagy Induction and Autophagosome Clearance in Neurons: Relationship to Autophagic Pathology in Alzheimer’s Disease	Barry Boland	Journal of Neuroscience	2008	811
2^nd^	mTOR signaling: At the crossroads of plasticity, memory and disease	Charles A. Hoeffer	Trends in Neurosciences	2010	789
3^rd^	Pten Regulates Neuronal Arborization and Social Interaction in Mice	Chang-Hyuk Kwon	Neuron	2006	692
4^th^	PTEN deletion enhances the regenerative ability of adult corticospinal neurons	Kai Liu	Nature Neuroscience	2010	652
5^th^	Loss of mTOR-Dependent Macroautophagy Causes Autistic-like Synaptic Pruning Deficits	Guomei Tang	Neuron	2014	599
6^th^	A synaptic trek to autism	Thomas Bourgeron	Current Opinion in Neurobiology	2009	465
7^th^	Rapamycin prevents epilepsy in a mouse model of tuberous sclerosis complex	Ling-Hui Zeng	Annals of neurology	2008	458
8^th^	Translational Regulatory Mechanisms in Persistent Forms of Synaptic Plasticity	Raymond J. Kelleher III	Neuron	2004	449
9^th^	Rapamycin causes regression of astrocytomas in tuberous sclerosis complex	David Neal Franz	Annals of neurology	2006	445
10^th^	Control of Dendritic Arborization by the Phosphoinositide-3b-Kinasee Phosphoinositides complextrocyynaptic Pl	Jacek Jaworski	Journal of Neuroscience	2005	439

## Discussion

Compared with traditional reviews, a bibliometric analysis allows for a more intuitive and scientific analysis of relevant papers in the field. Intuitive statistical charts and data analyses are beneficial for new researchers in this field. Bibliometrics is easier to understand than a meta-analysis. This can help young scholars easily grasp the overall trends of the field they want to study and accurately grasp the future study hot spots and development directions, thereby reducing unnecessary work. In addition, the current bibliometric analysis software is simple and easy to use.

According to the results of the bibliometric analysis, although the number of publications related to mTOR in neuroscience has declined slightly in the past few years, it has generally increased (Figures 3A,B), indicating that it remains a study hot spot, and mTOR-related publications may continue to increase in the next few years. A total of 3,029 manuscripts and 82,856 references were searched by the WoSCC. Among these 41 countries, studies conducted in the United States and China accounted for 47.4% of the total publications. As shown in Table 1, the United States ranked first in terms of the number of published articles, far exceeding that of other countries. Among the 10 organizations or institutions with the highest productivity, five were American institutions (Table 2). Although the number of articles published by Harvard University was not significantly different from that of Fudan University, the total number of citations by the top 10 Chinese organizations was far lower than that of Harvard University. These results show that the leading institutions for mTOR studies in neuroscience are mainly concentrated in European and American countries. Although the study field of neuroscience in China has developed rapidly and the gap between China and the United States has gradually narrowed, the quality of the article needs to be improved.

As can be observed from Table 4, Mustafa Sahin, from Harvard University, ranked first in the total number of citations, which shows that he has had a great influence on mTOR-related studies. Mustafa Sahin has published 19 articles related to mTOR in the scientific community. In 2019, Sahin’s team found that mTORC1 inhibitor (rapamycin) treatment significantly prolonged the survival time of Depdc5cc + mice and partially reduced attention deficit hyperactivity disorder, suggesting a new treatment strategy for clinical epilepsy (Yuskaitis et al., 2019). In 2018, Winden et al. (2018) summarized the relationship between mTOR and autism and proposed that mTOR inhibitors are expected to be an application prospect for improving neural development. Mustafa Sahin published a review named “The neurology of mTOR” in 2014; it is the first review to summarize the current situation and prospects of mTOR studies in neuroscience (Lipton and Sahin, 2014). The occurrence of AD is related to the enhancement of the mTOR signaling pathway (Van Skike et al., 2021). This is consistent with the results of the bibliometrics. Except in the field of neuroscience, the emerging scholar Zhang Xu’s team began to study mTOR in the field of ophthalmology, indicating that mTOR remains a concern for researchers in different clinical fields (Li et al., 2018; Wang et al., 2020).

Using CiteSpace’s clustering capabilities, the entire scientific field was divided into nine subtopics. As shown in Figure 6C, cluster 4 “ketamine,” cluster 5 “Alzheimer’s disease,” cluster 0 “spinal cord injury,” and cluster 3 “epilepsy” were significantly close together, indicating that ketamine plays a key role in AD, spinal cord injury (SCI), and epilepsy. Ketamine, an mTOR activator, exerts antidepressant effects (Zanos and Gould, 2018). Lozupone et al. (2018) explicitly discussed the possibility that ketamine may represent a potential therapy for AD, providing a new treatment strategy for AD. However, there is a lack of ongoing studies on ketamine in AD, and the side effects caused by new memory loss, confusion, separation, and psychosis remain unresolved (Smalheiser, 2019). Although acute administration of (R)-ketamine has a rapid antidepressant-like effect, it does not alter mTOR signaling (Yang et al., 2018). This may favor (R)-ketamine as the drug of choice for studies of AD with depression. However, the mechanism by which ketamine improves depressive symptoms is not fully understood, and the optimal dose, form, and route of administration have not yet been determined (Smalheiser, 2019). These problems are likely to become hot spots and directions for future studies. Fatma’s team found that ketamine has beneficial effects on pilocarpine-induced temporal lobe epilepsy in mice and that administration within minutes of seizures avoids nerve damage caused by pilocarpine (Tannich et al., 2020). However, the specific mechanism of epilepsy is unclear, and there is a lack of clinical studies on ketamine in epilepsy. This requires further exploration and excavation. Ketamine combined with environmental enrichment improves SCI prognosis in rats (Tai et al., 2021). SCI impairs mobility and often leads to complications, such as refractory neuropathic pain (Alcántar-Garibay et al., 2022). However, currently, the prognosis of SCI remains poor, and ketamine as a potential drug to improve SCI has recently become a concern (Tai et al., 2021).

As shown in Figure 6B, from 2015 to 2021, keywords, such as protein synthesis, messenger RNA, and translation were at the top of the list. mTOR transmits proliferative and anabolic signals to downstream transcriptional and translational devices, thereby regulating cellular energy production, protein synthesis, and autophagy (Kim et al., 2011; Saxton and Sabatini, 2017; Fidalgo da Silva et al., 2019; Kondo et al., 2021). The mTOR pathway is a complex network. Although the key players, such as 4E-BP, S6K, and LARP1, can explain the major phenomena observed in mTOR translation regulation, there are still some unresolved issues (Magnuson et al., 2012; Castelli et al., 2015; Philippe et al., 2020; Yang M. et al., 2022). For example, not all TOP mRNAs are controlled by 4E-BP, S6K, and LARP1 (Yang M. et al., 2022). This indicates that unknown proteins may be involved in this process. This may become a hot topic and a study direction in the future.

Several landmark papers were identified using the CiteSpace co-citation analysis (Figure 5C). Charles A Hoeffer published a review called “mTOR signaling: At the crossroads of plasticity, memory and disease,” with the strongest citation burst from 2011 to 2015. Hoeffer collected substantial evidence that mTOR signaling is associated with synaptic changes, memory, and neurological disorders (Tang and Schuman, 2002; Hoeffer and Klann, 2010; Rostamzadeh et al., 2019). However, little is known about the function of mTOR in other brain regions and its regulation in different classes of neurons (Hoeffer and Klann, 2010). At present, studies continue to focus on the pathogenesis of neurological diseases and new treatment strategies. Saxton and Sabatini (2017) reviewed recent advancements in mTOR function, regulation, and the importance of mammalian physiology and discussed the current and future prospects of clinically therapeutically targeted mTOR. These landmark articles provide readers with an authoritative understanding of the current mechanism of action of mTOR in neurological disorders and its potential applications.

Table 6 shows that the most studied neurological diseases are AD, TSC, and depression. AD is a chronic and progressive neurodegenerative disease of the brain and currently has no effective treatment (Sang et al., 2022). TSC is an autosomal dominant disorder that can cause disabling neurological disorders, such as epilepsy and TSC-related neuropsychiatric disorders (Luo et al., 2022). mTOR inhibitors (rapamycin/everolimus) show great potential in the treatment of TSCs, but there are two adverse effects that hinder their widespread use (Kingswood et al., 2021; Luo et al., 2022). Although classical antidepressants have been found to increase the expression and function of neurotrophic factors and activate mTOR, the pathogenesis of depression and the mechanism of action of current antidepressants are unclear (Ignácio et al., 2016). The most common studies of pathological mechanisms associated with mTOR are oxidative stress, neurodegeneration, ischemia, neuroinflammation, endoplasmic reticulum stress, and mitochondrial dysfunction. For example, several pieces of evidence have reported that stroke is associated with ischemia, neuroinflammation, and oxidative stress (Chamorro et al., 2016; Yan et al., 2022; Yang Y.D. et al., 2022). Hou et al. (2018) found resveratrol provides neuroprotection by activating the JAK2/STAT3/PI3K/AKT/mTOR pathways in stroke rat models. In addition, Chen et al. (2018) found that ginsenoside Rg1 alleviates cognitive impairment and neural senescent stem cells in D-galactose-induced mouse models by reducing oxidative stress and downregulating Akt/mTOR signaling. These phenomena show that the current hot spot is still the pathogenesis of neurological diseases and new treatment strategies.

This can provide future study directions for new researchers who have recently entered this field of study.

## Limitations

Although this is the first bibliometric study of mTOR in neuroscience, there are some limitations that have been reported in other bibliometric study papers (Chen et al., 2020; Landucci et al., 2021; Ma et al., 2021). The deadline for publication of this study is 31 December 2021, but the WoSCC will be updated constantly. Some kinds of literature have been found online in 2022; therefore, this part is omitted in this article. In addition, only publications with the term “mTOR” or “mammalian target of rapamycin” in their titles, abstracts, and keywords were recovered. However, studies with these terms in the text were not included in the analysis. In addition, some documents that were not included in the WoSCC were not considered because the search was limited to WoSCC-indexed journals. Although there are some limitations in the bibliometric study on mTOR in neuroscience, the results of this study are stable because this study now covers most papers from 2002 to 2021, and newly published papers will not affect the final results.

## Conclusion

By bibliometric scientific analysis, future study hot spots will focus on protein synthesis regulation, ischemia, mitochondrial dysfunction, oxidative stress, neuroinflammation, AD, TSC, and depression. This can provide future study directions for researchers who have recently entered this field.

## Data availability statement

The original contributions presented in this study are included in the article/supplementary material, further inquiries can be directed to the corresponding author.

## Author contributions

LL and LS designed the study. LL, XX, YL, YZ, XL, and BY collected and analyzed the experimental data and prepared the figures. LL wrote the manuscript. LS and YL revised the manuscript for intellectual content. All authors approved the final version of this manuscript.

## Footnotes

1
https://sourceforge.net/projects/citespace/files/latest/download

2
https://www.vosviewer.com/download#downloadvosviewer
